# An Unusual Presentation of Oral Mucocele in Infant and Its Review

**DOI:** 10.1155/2014/723130

**Published:** 2014-08-26

**Authors:** Neha Bhargava, Prateek Agarwal, Nitin Sharma, Mayank Agrawal, Mohsin Sidiq, Pooja Narain

**Affiliations:** ^1^Department of Pediatric and Preventive Dentistry, Rajasthan Dental College and Hospital, N.H. 08, Bagru Khurd, Ajmer Road, Jaipur, Rajasthan 302026, India; ^2^Department of Oral and Maxillofacial Surgery, Mahatma Gandhi Dental College and Hospital, Sitapura, Jaipur, Rajasthan 302022, India; ^3^Department of Preventive and Community Dentistry, Rajasthan Dental College and Hospital, N.H. 08, Bagru Khurd, Ajmer Road, Jaipur, Rajasthan 302026, India; ^4^Department of Pediatric and Preventive Dentistry, Government Dental College and Hospital, Shireen Bagh, Kak Sarai, Srinagar, Kashmir 190010, India; ^5^Department of Oral and Maxillofacial Pathology, Rajasthan Dental College and Hospital, N.H. 08, Bagru Khurd, Ajmer Road, Jaipur, Rajasthan 302026, India

## Abstract

Mucocele is a benign lesion characterized by an extravasation or retention of mucous in submucosal tissue from minor salivary glands. Mucoceles are known to occur most commonly on the lower lip, followed by the floor of mouth and buccal mucosa being the next most frequent sites. Trauma and lip biting habits are the main cause for these types of lesions. Mucocele is a common oral mucosal lesion but it is rarely observed in the infant. This paper highlights the successful management of a rare case of mucocele in an 11-month-old child. Diagnosis and management of mucocele are challenging. For this reason we felt it would be interesting to review the clinical characteristics, histological features, differential diagnosis, and their treatment and evolution in order to aid decision-making in daily clinical practice.

## 1. Introduction

Oral mucocele represents one of the most common benign lesion of the oral mucosa that means a cavity filled with mucus (muco means mucus and coele means cavity), which is the secretory product of salivary glands. The mechanisms for the development of these lesions are two, mucus extravasation, generally regarded as being of traumatic origin, and mucus retention, resulting from obstruction of the duct of a minor or accessory gland. When located on the floor of the mouth these lesions are called ranulas because the inflammation resembles the cheeks of a frog [1]. The most common site of occurrence of mucocele is the lower lip, the lesion has no sex predilection, and all age groups are susceptible, with the peak frequency reported to be in the second and third decades and rarely observed in infants making the diagnosis and management of mucocele challenging [2]. Mucocele has clinical resemblance with many other swellings and ulcerative lesions of oral cavity and hence needs to be differentiated carefully. Here we report an interesting unusual case of mucocele of the lower lip in an infant, along with emphasis given on its etiopathogenesis, clinical presentation, and various treatment modalities.

## 2. Case Report

**Figure 1:**
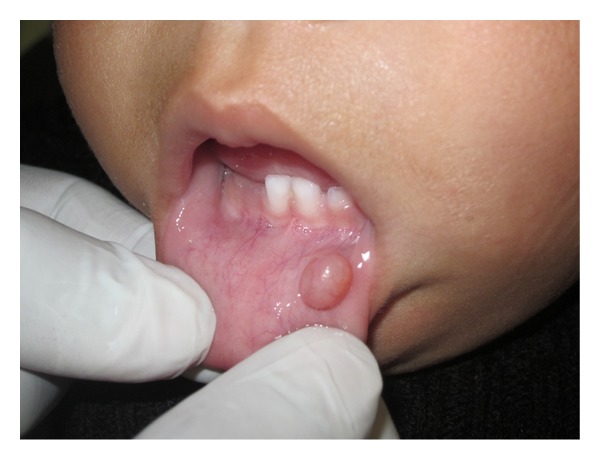
Mucocele in the lower lip of baby at 11 months.

**Figure 2:**
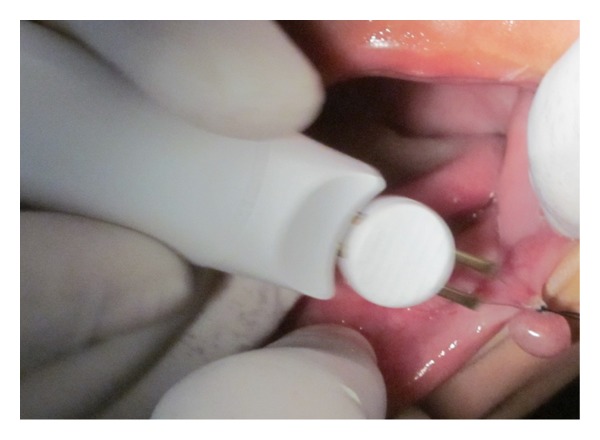
Excision of the lesion using electrocautery.

**Figure 3:**
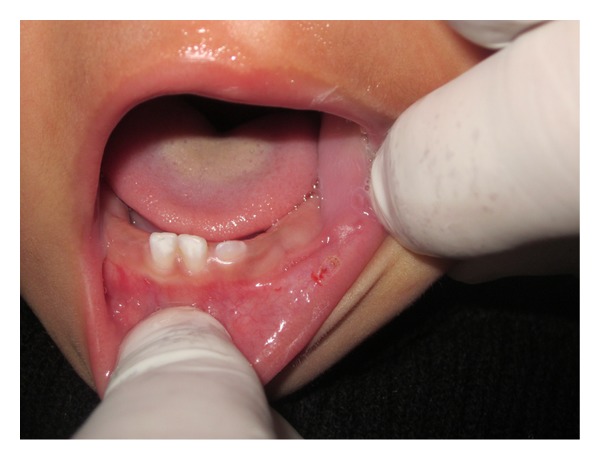
Immediate postoperative view.

**Figure 4:**
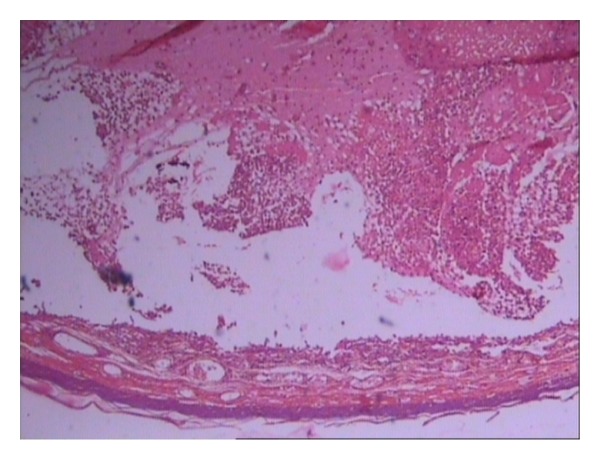
H&E stained section reveals stratified squamous epithelium with underlying connective tissue consisting of large central mucin pooled area surrounded by granulation tissue and chronic inflammatory cells.

An 11-month-old male patient was referred to our department with the chief complaint of a “little ball” in the lower lip and that he had difficulty in sucking for more than 3 months. The baby was in good general health and no other symptoms were reported. Oral habits or a local trauma was not reported. The clinical examination revealed the presence of a soft tissue nodule on the lower lip mucosa (Figure 1) which was similar in color to the oral mucosa measuring approximately 5 cm at its widest diameter with a sessile base, flaccid consistency, clearly defined limits, and a smooth surface. Based on detailed history and clinical examination a provisional diagnosis of mucocele was made. After medical evaluation, and signed informed consent from the parents, an excisional biopsy was performed under local anesthesia. Due to the lack of baby's contribution, considering his little age, and as the procedure was simple, a decision was taken in favor of the physical containment (protective stabilization) with consent and aid of the parents: laying the baby on the chair, the mother laying over him holding the hands, and the assistant holding the baby's head. As the baby was crying continuously, it helped in keeping the mouth open. A local infiltrative anesthesia (2% lignocaine with epinephrine 1 : 80,000; one cartridge) was infiltrated around the lesion. Before infiltration, a topical anesthetic gel for 2 minutes was applied. The lip was then everted with digital pressure to increase the lesion's prominence. A thick silk thread was passed through the lesion at its largest diameter and a surgical knot was made followed by excisional biopsy using electrocautery (Figures 2 and 3), hence minimizing the chances of pain and postoperative bleeding. An analgesic was prescribed on the first postoperatory day to prevent any possible pain that could result in stress for the baby. The specimen was sent for histopathologic analysis which identified a large central mucous pooled area consisting of mucinophages, mucin containing cells, surrounded by compressed connective tissue wall, and forming granulation tissue (Figure 4) and confirmed the diagnosis as mucocele. After 2 hours, the patient recovered normal breastfeeding. The child reported uneventful recovery and an improved dietary habit one week postoperatively.

**Figure 5:**
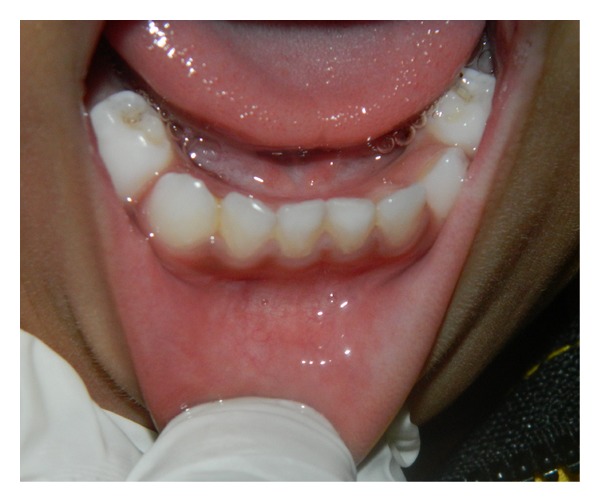
Appearance of the surgical area 12 months after the intervention, no recurrence.

The baby was reexamined after 15 days and 6 and 12 months. No recurrence was observed after 12 months (Figure 5).

## 3. Discussion

Yamasoba et al. [3] highlighted two crucial etiological factors in mucoceles as follows:

- trauma,
- obstruction of salivary gland duct.

Mainly physical trauma causes a spillage of salivary secretion into surrounding submucosal tissue. Later inflammation may become obvious due to stagnant mucous. Habit of lip biting and tongue thrusting are also one of the aggravating factors [4].

The extravasation type will undergo three evolutionary phases [5].

- In the first phase there will be spillage of mucus from salivary duct into the surrounding tissue in which some leucocytes and histiocytes are seen.
- In second phase, granulomas will appear due to the presence of histiocytes, macrophages, and giant multinucleated cells associated with foreign body reaction. This second phase is called as resorption phase.
- Later in the third phase there will be a formation of pseudocapsule without epithelium around the mucosa due to connective cells.

The retention type of mucocele is commonly seen in major salivary glands. It is due to the dilatation of duct due to block caused by a sialolith or dense mucosa [5]. It depends upon the obstruction of salivary flow from secretory apparatus of the gland.

### 3.1. Clinical Characteristics

Clinically they are characterized by single or multiple, spherical, fluctuant nodules, ranging from normal pink to deep blue in color, and are generally asymptomatic. The tissue cyanosis and vascular congestion associated with stretched overlying tissue and the translucency of the accumulated fluid beneath result in the deep blue color. At times it may rupture leaving slightly painful erosions that usually heal within few days. Para functional habits such as lip biting and Lip sucking and trauma explain the lower lip being the most commonly described location of extravasation mucoceles [6]. They are mainly found in children and young patients with equal incidences in both sexes and rarely seen among children less than one year of age.

### 3.2. Diagnosis

The history and clinical findings lead to the diagnosis of a superficial mucocele. The appearance of mucocele is pathognomonic and the following data are crucial: lesion location, history of trauma, rapid appearance, variations in size, bluish color, and the consistency [7]. Usually mucoceles are mobile lesions with soft and elastic consistency depending on how much tissue is present over the lesion. Despite this fluctuation, a drained mucocele would not fluctuate and a chronic mucocele with a developed fibrosis would have less fluctuation. In retention type mucoceles, cystic cavity with well-defined epithelial wall lined with cuboidal cells is present. This type shows less inflammatory reaction. The extravasation type is a pseudocyst without epithelial wall and shows inflammatory cells and granulation tissues. Even though there is no epithelial covering around the mucosa, this is well encapsulated [4].

Radiographs are the contributing factors in diagnosis of ranulas. Localization of these lesions is done by computed tomography and magnetic resonance imaging. High amylase and protein content can be revealed by the chemical analysis [8]. A histopathologic study is crucial to confirm the diagnosis which shows the presence of ductal epithelium, granulation tissue, pooling of mucin, and inflammatory cells.

Mucocele has clinical resemblance with many other swellings and ulcerative lesions of oral cavity and hence needs to be differentiated carefully. Palpation can be helpful for a correct differential diagnosis. Lipomas and tumors of minor salivary glands present no fluctuation while cysts, mucoceles, abscess, and hemangiomas do. A simple technique known as fine needle aspiration biopsy (FNAB) is very helpful, especially when differential diagnosis of angiomatous lesions is involved [5]. Here we have attempted to list the probable differential diagnosis of mucocele occurring at most common site, that is, lower lip, along with all the clinical features that helps in their differentiation (Table 1).

### 3.3. Treatment

Conventional surgical removal is the most common method used to treat this lesion. Other treatment options include CO_2_ laser ablation, cryosurgery, intralesional corticosteroid injection, micromarsupialization, marsupialization, and electrocautery [9].

There is no difference in the treatment of retention and extravasation mucocele. Small sized mucoceles are removed with marginal glandular tissue and in case of large lesions marsupialization will help to avoid damage to vital structures and decrease the risk of damaging the labial branch of mental nerve [9]. Lacrimal catheters are used to dilate the duct to remove the obstruction of retention type mucoceles. While removing the mucocele surgically, remove the surrounding glandular acini, remove the lesion down to the muscle layer, and avoid damage to the adjacent gland and duct while placing the suture, as these are some strategies to reduce recurrence. If the fibrous wall of the mucocele is thick, then the removed tissue must be sent for histopathological examination to rule out any salivary gland neoplasms [10]. The micromarsupialization can be considered as an alternative treatment in case of pediatric patient because this technique is simple, relatively painless, and of less chances of recurrence. This technique (after disinfection and anaesthesia) consists of passing thick silk thread through the lesion at its largest diameter and then making a surgical knot. The suture is removed after 7–10 days, enough time for the mucocele to disappear [5]. The advantage in CO_2_ laser is that it minimizes the recurrences and complications and allows rapid, simple mucocele ablation. It is also indicated for the patients who cannot tolerate long procedures [9]. Other therapies that are of less well-proved efficacy include intralesional corticosteroid injections and gamma-linolenic acid. These therapies are of importance particularly in cases of multiple mucoceles, where surgical dissection of each lesion becomes difficult [6].

## 4. Conclusion

Mucocele is the most common benign self-limiting condition. Since these lesions are painless, it is the dentists, who usually pick up these lesions when the patient comes for a routine oral check or an unrelated dental problem. Management of mucocele becomes challenging because of their high chances of recurrence. However, surgical excision with dissection of surrounding and contributing minor salivary glands proved to be successful with least recurrence.

## Figures and Tables

**Table 1:** Differential diagnosis of mucocele occurring on most common site, lower lip.

Lesion	Age	Sex	Site	Clinical appearance	Consistency	Progression
Fibroma	Common in 3rd, 4th, and 5th decade	M : F = 1 : 2	Common on labial mucosa	Elevated, smooth surfaced, sessile, or pedunculated nodule of normal pink color. Usually small to rarely several cm in size	Firm	Slowly growing

Lipoma	Usually in 4th decade	M : F = 1 : 1	Less common on lower lip	Smooth surfaced, yellowish, sessile or pedunculated, asymptomatic, nodular mass. Usually less than 3 cm in size	Soft and freely movable	Slowly growing

Hemangioma	Infancy	M : F = 1 : 3	Lip is a common site	Flat or raised, deep red or bluish red, and seldom well circumscribed	Readily compressible, blanch and filling slowly when released	Rapidly growing for initial 6–10 months and then slowing in growth and involute

Varix	Older adults		Lip is a common site	Asymptomatic, nontender, bluish-purple nodule	Firm	

Traumatic neuroma	Middle aged adults	Slightly more common in females	Lower lip is a common site	Smooth surfaced, nonulcerated nodule of normal color with history of trauma. Usually less than 1 cm	Digital pressure may cause considerable pain	Slowly growing

Salivary duct cyst	Adults		Lip is a common site	Smooth surfaced, bluish swelling	Soft and fluctuant	Slowly growing

Epidermoid cyst	3rd and 4th decade	M : F = 2 : 1	Lip is a fairly common site	Painless, round, flesh colored to yellowish-white nodule present midline	Firm and mobile	Slowly growing

Mucoepidermoid carcinoma	2nd to 7th decade	Slight female predilection	Lower lip is a common site	Low grade tumor appears as a painless mass seldom exceeding 5 cms in diameter. High grade tumor produces pain, numbness, and ulceration. Minor salivary gland tumors have blue or red color	Low grade is usually soft and fluctuant, while high grade tumor is firm	Low grade tumor slowly enlarging, while high grade tumor rapidly enlarging

Amelanotic or blue nevi	Usually in younger patients	Predominant in women	Labial mucosa is a fairly common site	Asymptomatic, round or oval, raised or slightly raised, and sessile growth of normal or blue-black color	Soft to firm	Slowly growing

Granular cell tumor	4th to 6th decade of life	M : F = 1 : 2	Lip is a less common site	Asymptomatic, sessile, pink or yellowish nodular mass	Firm and immovable	

Lymphangioma	Usually present at birth	M : F = 1 : 1	Lip is a less common site	Asymptomatic tumor mass of pink or purple color with pebbled surface	Soft	

Pyogenic granuloma	Mostly in children and young adults	Female predilection	Lip is a fairly common site	Smooth, pedunculated or sessile, pink to red to purple colored, few mms to several cm in size, and painless swelling	Soft	May exhibit rapid growth
